# Evaluation of COVID-19 Mitigation Policies in Australia Using Generalised Space-Time Autoregressive Intervention Models

**DOI:** 10.3390/ijerph18147474

**Published:** 2021-07-13

**Authors:** Ryan H. L. Ip, Dmitry Demskoi, Azizur Rahman, Lihong Zheng

**Affiliations:** School of Computing and Mathematics, Charles Sturt University, Wagga Wagga, NSW 2650, Australia; ddemskoy@csu.edu.au (D.D.); azrahman@csu.edu.au (A.R.); lzheng@csu.edu.au (L.Z.)

**Keywords:** delay effects, feasible GLS, GSTARX, multiple interventions, policy evaluation

## Abstract

In handling the COVID-19 pandemic, various mitigation policies aiming at slowing the spread and protecting all individuals, especially the vulnerable ones, were implemented. A careful evaluation of the effectiveness of these policies is necessary so that policy-makers can implement informed decisions if another wave of COVID-19 or another pandemic happens in the future. This paper reports an assessment of some policies introduced by the Australian governments using a generalised space-time autoregressive model which incorporates multiple exogenous variables and delay effects. Our results show that the number of daily new cases from the states and territories are influenced by both temporal and spatial aspects. Business and border restrictions are found helpful in reducing the number of new cases a few days after implementation while gathering restrictions may not be effective.

## 1. Introduction

As of 22 April 2021, the unprecedented COVID-19 pandemic has led to over three million deaths around the globe [1]. As the virus is highly contagious, various mitigation policies including, but not limited to, travel restrictions, cancellation of mass gathering events, mandatory quarantine, shut downs of restaurants and businesses, stay-at-home orders and mandatory face covering [2] were implemented. Although different countries may utilise different methods at different timings, the common goal is to slow down the spread aiming at protecting all individuals and relieving pressure on the healthcare system until effective antiviral drugs or vaccines become widely available [3,4]. Currently, it is uncertain how long these mitigation policies should remain in place due to the uncertainties regarding the timing of an effective vaccine and the evolutionary dynamics of the virus. While these policies may be necessary, the socioeconomic consequences cannot be ignored. Although the full economic impact remains unclear, it was predicted that a lockdown of Tokyo would lead to a 5.3% loss of Japan’s annual GDP [5]. Similarly, the authors of [6] estimated a decline of 5% in the real GDP growth for each month under partial economic shutdown in the United States. Apart from the economic impacts, mitigation policies have also affected the mental health of people. For example, the authors of [7] reported a number of suicide cases related to lockdown and economic factors in Bangladesh. Furthermore, the authors of [8] reported associations between adverse mental health outcomes in the general population in Italy and the lockdown measures. A narrative review conducted in [9] showed that measures such as school closures had resulted in a negative impact on the mental health among children and adolescents. Therefore, it is necessary to evaluate the effectiveness of the policies implemented for future planning and policy review so that informed decisions can be made in case of another wave of COVID-19 or another pandemic.

In Australia, the first case of COVID-19 was reported on 25 January 2020 [10]. On 27 February, Prime Minister Scott Morrison announced the activation of the Australian Health Sector Emergency Response Plan [11]. A human biosecurity emergency was declared on 18 March [12]. Subsequently, Australia announced to close its border on 20 March [13]. Additional restrictions and guidelines for managing COVID-19 were introduced by the Federal government in late March but state and territory level governments were allowed to implement them differently [14]. State and territory level policies included economic packages and interstate border restrictions [15]. Some of these policies will be reviewed in more details in Section 2.2. Due to the timing, intensity and number of policies implemented by the government of each state, the effectiveness of the policies may also differ from state to state.

The daily numbers of new cases recorded from the states can be considered as multiple time series that are spatially correlated. To properly assess the efficacy of the mitigation policies, a stochastic model that takes into account both spatial and temporal information, and allows the incorporation of exogenous variables, is required. We briefly review some of the previously proposed space-time models and their limitations. Autoregressive moving average (ARMA) models have long been used in analysing time series data [16]. When it is required to analyse multiple time series jointly, it can be done through vector autoregressive moving average (VARMA) models [17]. A clear drawback of these models is the inability to capture the spatial dependencies between the time series. Space-time autoregressive moving average (STARMA) models [18,19] extend the ARMA models by incorporating spatial information. Results regarding the estimation of STARMA models can be found in [20]. In this class of models, the autoregressive parameters are assumed to be fixed across all locations, which may not be desirable [21]. The generalised space-time autoregressive moving average (GSTARMA) models examined by the authors of [22] relax such restrictive assumption to allow site-specific autoregressive parameters. The authors of [23] reported an application of the GSTAR model on a set of GDP data collected across Europe. Note that all these models do not depend on exogenous variables. To allow prediction based on the levels of some explanatory variables, the authors of [24] introduced the STARMAX (STARMA with eXogenous variables) model, while the authors of [25] proposed the GSTARX models.

Any intervention such as an implementation of a policy can be considered as an exogenous variable when coded as an indicator variable. Time series intervention models [16,26] have been used in assessing the effectiveness of policies in various contexts, including imperfectly identifiable natural events [27], river monitoring [28], marine pollution monitoring [29] as well as public health interventions [30]. Intervention analysis is less commonly used in the spatio-temporal setting, with an exception of the authors of [25] who studied the time series of oil price in four cities with Eid (a religious festival celebrated by Muslims) considered as the intervention. In this work, we propose to use a more general modelling framework compared to the one used in [25]. The key differences between our work and theirs are (a) we use multiple interventions, as opposed to the single one; (b) while their intervention applies to all sites at the same time, our interventions may apply to each of the sites at different times; and (c) delay effects of exogenous variables are taken into account in our work as well.

The rest of the paper is organised as follows. Section 2 details the data used and the policies considered in our analysis. Section 3 describes the GSTARX model, which will be used to assess the effectiveness of the mitigation policies implemented in Australia and the estimation procedure. The results are presented in Section 4. Some concluding remarks are provided in Section 5.

## 2. Data

### 2.1. Number of COVID-19 Cases in Australia

**Figure 1:**
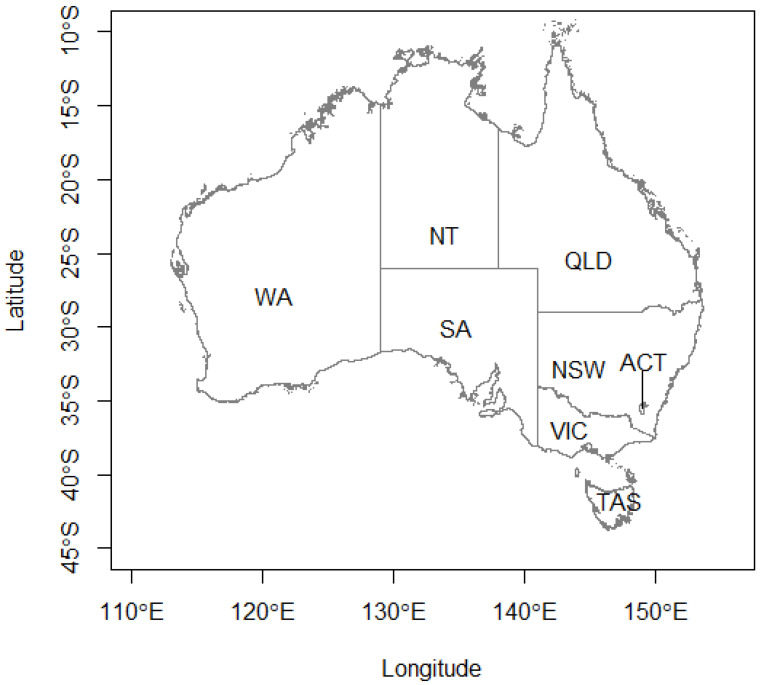
Map of Australia with the states and territories indicated.

The daily cumulative number of confirmed COVID-19 cases from eight states and territories, namely, Australian Capital Territory (ACT), New South Wales (NSW), Northern Territory (NT), Queensland (QLD), South Australia (SA), Tasmania (TAS), Victoria (VIC) and Western Australia (WA), formed the backbone of the dataset. Figure 1 shows a map of Australia with the states and territories indicated. Note that ACT is completely surrounded by NSW. Data from 25 January 2020 to 12 September 2020 were obtained from the webpage of the Department of Health, Australia Government [10]. Daily number of new cases were found from these cumulative numbers. Correspondingly, the data consist of eight spatially dependent time series (N=8), each represents a state or territory with a length of 232 days (T=232).

On ten occasions, a negative number of new cases was observed. We conjecture that these negative numbers may be due to clerical errors or false positive cases recorded in previous days. Without any information regarding the true reasons, these ten negative numbers were replaced by zeros. One was added to these numbers to make sure there were no zero entries, so that logarithms can be applied later on. Next, the numbers were divided by the population of the respective state or territory on 31 March 2020 reported by the Australian Bureau of Statistics [31] for a fair comparison across the states. Natural logarithm was then applied to achieve approximate normality. Mathematically, let ${\tilde{Z}}_{i}\left(t\right)$ be the original number of new cases recorded on day *t* in state or territory *i*. We worked with the transformed variable

(1) $$Z_{i}\left(t\right)=\log\left(\frac{\max\{{\tilde{Z}}_{i}\left(t\right),0\}+1}{P_{i}}\right),$$

where $P_{i}$ is the population of state or territory *i*. Each time series of the transformed variable was standardised by subtracting the mean and further divided by the standard deviation to stabilise the variance. In other words, the standardised logarithm of the population-adjusted daily number of new cases was used as the response variable.

### 2.2. Policies

Among all policies implemented, we selected three broad categories, which are Gathering (*G*), Economy (*E*) and Border control (*B*), for a more in-depth analysis. As various restrictions and rules were imposed at different strictness across the states and territories, it is difficult to compare them exactly. We attempted to define the levels within these categories according to the common features of the restrictions imposed. The descriptions of these levels can be found in Table 1. All policies and implementation details were found from the webpages of the corresponding authorities: ACT Government [32], NSW Health [33], NT Government [34], QLD Government [35], SA Health [36], TAS Government [37], Department of Health and Human Services [38] and WA Government [39].

The variable Gathering (*G*) is based on the restrictions on public gatherings that include gathering in parks and public events such as music festivals, with limited exemptions such as weddings and funerals. In each state or territory, the notion of “social distancing” (e.g., 1.5 m apart) has remained as the time the restrictions were introduced. Although the definition varied at certain times (e.g., changing from 1.5 m to a certain number of people within a particular area), these rules were too difficult to measure and enforce in reality. Thus, we did not include them as separate variables in our analysis. Based on the upper limit on the number of people allowed for a public gathering, four levels were defined (Table 1). Accordingly, three indicator variables were defined: $G_{i}=1$ if the *i*-th level gathering restriction was in place, and 0 otherwise, for i=1,2,3.

The variable Economy (*E*) is based on the restrictions imposed on businesses and other service providers. Restrictions were interpreted differently by the various states and territories, so it is not easy to compare their levels exactly. Commonly, there were four levels as listed in Table 1 where the descriptions were mainly based on the interpretations used by Federal, NSW, VIC and QLD Governments. Restrictions set by other jurisdictions have been fitted to these in the closest way possible. Accordingly, three indicator variables were defined: $E_{i}=1$ if the *i*-th level economy restriction was in place, and 0 otherwise, for i=1,2,3.

The variable Border Control (*B*) is based on the closures of international and interstate borders. Some states and territories took action to protect themselves while some did not, so a border closure may have increased the level for one state or territory but not its neighbour. For example, when the second wave hit Victoria in mid-July, New South Wales closed its border with Victoria. However, Victoria did not close the same border. As shown in Table 1, Level 1 border control was referring to the closure of the international border while Levels 2 and 3 depended on the interstate borders. Accordingly, three indicator variables were defined: $B_{i}=1$ if the *i*-th level border control was in place, and 0 otherwise, for i=1,2,3.

**Figure 2:**
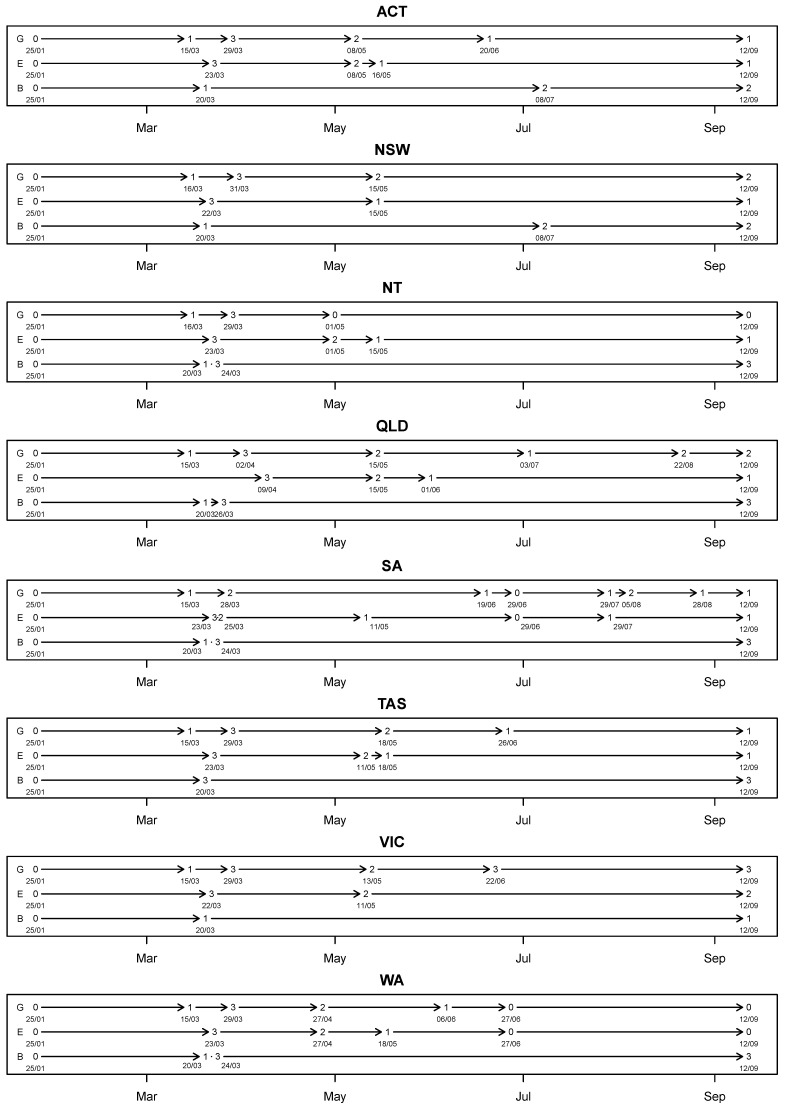
Dates and levels of the policies implemented in each of the states and territories.

Altogether, nine exogenous variables, each being an indicator variable, were considered in our application. Figure 2 provides the timeline which shows the dates and levels of policies implemented in each of the states and territories.

## 3. Methods

This section provides the details of model used in our analysis, including the the specification of the spatial weight matrix, the structure of the covariates and the estimation procedures.

### 3.1. The GSTARX Model

We formalise the idea in this section. Denote by $Z\left(t\right)={\left[\begin{array}{c} Z_{1}\left(t\right),Z_{2}\left(t\right),\ldots ,Z_{N}\left(t\right) \end{array}\right]}^{⊤},\;t=1,2,\ldots ,T,$ an *N* dimensional random process at time *t*, where the subscripts i=1,2,…,N specify the location where $Z_{i}\left(t\right)$ will be observed. Throughout the discussions below, we assume *N* is fixed, meaning that the number of sites where Z is observed remains the same throughout the period considered. The generalised space-time autoregressive model with autoregressive order *p*, spatial orders $\lambda =(\lambda_{1},\lambda_{2},\ldots ,\lambda_{p})$ and delay effects $d=(d_{1},d_{2},\ldots ,d_{m})$, abbreviated as GSTARX(p;λ;d), defined in [25], can be written as

(2) $$Z\left(t\right)=\sum_{k=1}^{p}\sum_{\ell =0}^{\lambda_{k}}\Phi_{k\ell }W^{\left(\ell \right)}Z\left(t-k\right)+\sum_{q=1}^{m}X_{q}\left(t-d_{q}\right)\beta_{q}+\varepsilon \left(t\right),$$

where

- *p* is the autoregressive order,
- $\lambda_{k}$ is the spatial order for *k*-th autoregressive term,
- $W^{\left(\ell \right)}$ is an N×N weight matrix which specifies the *ℓ*-th order spatial weights (see Section 3.3 for further details),
- $\Phi_{k\ell }$ is an N×N diagonal matrix with elements $\phi_{k\ell }^{\left(1\right)},\phi_{k\ell }^{\left(2\right)},\ldots ,\phi_{k\ell }^{\left(N\right)}$ where each $\phi_{k\ell }^{\left(\;\cdot \;\right)}$ is an autoregressive parameter to be estimated,
- $X_{q}\left(t-d_{q}\right)$ is a N×N diagonal matrix with the *i*-th diagonal element being $X_{iq}\left(t-d_{q}\right)$ representing the *q*-th exogenous variable observed at time $t-d_{q}$ at location *i*,
- $\beta_{q}={\left[\begin{array}{c} \beta_{q}^{\left(1\right)},\beta_{q}^{\left(2\right)},\ldots ,\beta_{q}^{\left(N\right)} \end{array}\right]}^{⊤}$ is the vector of the coefficients associated with the *q*-th exogenous variable, and
- ε(t) represents the random error terms, assumed to follow the *N*-dimensional multivariate normal distribution with a mean of 0 and a covariance matrix Σ.

If there are reasons to believe that the effects of the exogenous variables are the same across all locations, the GSTARX model can be written as

(3) $$Z\left(t\right)=\sum_{k=1}^{p}\sum_{\ell =0}^{\lambda_{k}}\Phi_{k\ell }W^{\left(\ell \right)}Z\left(t-k\right)+X\left(t;d\right)\beta +\varepsilon \left(t\right),$$

where X(t;d) is a N×m matrix such that

(4) $$X\left(t;d\right)=\left[\begin{array}{cccc} X_{11}\left(t-d_{1}\right) & X_{12}\left(t-d_{2}\right) & \cdots & X_{1m}\left(t-d_{m}\right)\\ \vdots & \vdots & \ddots & \vdots \\ X_{N1}\left(t-d_{1}\right) & X_{N2}\left(t-d_{2}\right) & \cdots & X_{Nm}\left(t-d_{m}\right) \end{array}\right]$$

and $\beta ={\left[\begin{array}{c} \beta_{1},\beta_{2},\ldots ,\beta_{m} \end{array}\right]}^{⊤}$ is the vector of the coefficients.

Both (2) and (3) are written from the cross-sectional standpoint. When data from all t=1,2,…,T are incorporated, one could “vectorise” the responses location-by-location. As a result, the model can equivalently be represented in a more compact manner. For example, consider N=T=3, a GSTARX(1;1;(1,0)) with two exogenous variables admits the form

$$\begin{array}{c} \left\{\begin{array}{c} Z_{1}\left(t\right)=\phi_{10}^{\left(1\right)}Z_{1}\left(t-1\right)+\phi_{11}^{\left(1\right)}\sum_{j\neq 1}w_{1j}^{\left(1\right)}Z_{j}\left(t-1\right)+\beta_{1}^{\left(1\right)}X_{11}\left(t-1\right)+\beta_{2}^{\left(1\right)}X_{12}\left(t\right)+\varepsilon_{1}\left(t\right),\\ Z_{2}\left(t\right)=\phi_{10}^{\left(2\right)}Z_{2}\left(t-1\right)+\phi_{11}^{\left(2\right)}\sum_{j\neq 2}w_{2j}^{\left(1\right)}Z_{j}\left(t-1\right)+\beta_{1}^{\left(2\right)}X_{21}\left(t-1\right)+\beta_{2}^{\left(2\right)}X_{22}\left(t\right)+\varepsilon_{2}\left(t\right),\\ Z_{3}\left(t\right)=\phi_{10}^{\left(3\right)}Z_{3}\left(t-1\right)+\phi_{11}^{\left(3\right)}\sum_{j\neq 3}w_{3j}^{\left(1\right)}Z_{j}\left(t-1\right)+\beta_{1}^{\left(3\right)}X_{31}\left(t-1\right)+\beta_{2}^{\left(3\right)}X_{32}\left(t\right)+\varepsilon_{3}\left(t\right), \end{array}\right. \end{array}$$

for t=2 and 3. If we stack the equations by location, the model can be written as

(5) Z=YΦ+ε,

where

(6) $$\begin{array}{ccc} Z & = & {\left[\begin{array}{cccccc} Z_{1}\left(2\right) & Z_{1}\left(3\right) & Z_{2}\left(2\right) & Z_{2}\left(3\right) & Z_{3}\left(2\right) & Z_{3}\left(3\right) \end{array}\right]}^{⊤},\\ Y & = & \left[\begin{array}{cccccc} Z_{1}^{*} & 0 & 0 & X_{1}\left(d\right) & 0 & 0\\ 0 & Z_{2}^{*} & 0 & 0 & X_{2}\left(d\right) & 0\\ 0 & 0 & Z_{3}^{*} & 0 & 0 & X_{3}\left(d\right) \end{array}\right],\\ Z_{i}^{*} & = & \left[\begin{array}{cc} Z_{i}\left(1\right) & \sum_{j\neq i}w_{ij}^{\left(1\right)}Z_{j}\left(1\right)\\ Z_{i}\left(2\right) & \sum_{j\neq i}w_{ij}^{\left(1\right)}Z_{j}\left(2\right) \end{array}\right]\\ X_{i}\left(d\right) & = & \left[\begin{array}{cc} X_{i1}\left(1\right) & X_{i2}\left(2\right)\\ X_{i1}\left(2\right) & X_{i2}\left(3\right) \end{array}\right],\\ \Phi & = & {\left[\begin{array}{cccccccccccc} \phi_{10}^{\left(1\right)} & \phi_{11}^{\left(1\right)} & \phi_{10}^{\left(2\right)} & \phi_{11}^{\left(2\right)} & \phi_{10}^{\left(3\right)} & \phi_{11}^{\left(3\right)} & \beta_{1}^{\left(1\right)} & \beta_{2}^{\left(1\right)} & \beta_{1}^{\left(2\right)} & \beta_{2}^{\left(2\right)} & \beta_{1}^{\left(3\right)} & \beta_{2}^{\left(3\right)} \end{array}\right]}^{⊤},\\ \varepsilon & = & {\left[\begin{array}{cccccc} \varepsilon_{1}\left(2\right) & \varepsilon_{1}\left(3\right) & \varepsilon_{2}\left(2\right) & \varepsilon_{2}\left(3\right) & \varepsilon_{3}\left(2\right) & \varepsilon_{3}\left(3\right) \end{array}\right]}^{⊤}\;∼\;N_{6}\left(0,I_{2}⊗\Sigma \right). \end{array}$$

As this representation is analogous to a usual linear model, the parameters can be estimated using ordinary least squares method as long as Y has the full rank. The estimator can be expressed as

(7) $${\hat{\Phi }}_{\mathrm{OLS}}={\left(Y^{\prime }Y\right)}^{-1}Y^{⊤}Z.$$

Occasionally the covariates may make the rank of Y less than full. For example, in the context of intervention analysis, some policies may not have been implemented in some locations throughout the period of study. As a result, there will be a column of zeros in Y. Pragmatic approaches could be applied to fix the issue. For example, one may redefine the variables by collapsing some of the levels. Another quick remediation is to employ representation (3) rather than (2) by assuming the effects of exogenous variables are the same across all locations. Consequently, $\beta_{q}^{\left(1\right)}=\beta_{q}^{\left(2\right)}=\cdots =\beta_{q}^{\left(N\right)}=\beta_{q}$ for all q=1,2,…,m, and Y, and Φ in (6) has to be modified as:

(8) $$\begin{array}{ccc} Y & = & \left[\begin{array}{cccc} Z_{1}^{*} & 0 & 0 & \\ 0 & Z_{2}^{*} & 0 & X(d)\\ 0 & 0 & Z_{3}^{*} & \end{array}\right],\\ X(d) & = & \left[\begin{array}{cc} X_{11}\left(1\right) & X_{12}\left(2\right)\\ X_{11}\left(2\right) & X_{12}\left(3\right)\\ X_{21}\left(1\right) & X_{22}\left(2\right)\\ X_{21}\left(2\right) & X_{22}\left(3\right)\\ X_{31}\left(1\right) & X_{32}\left(2\right)\\ X_{31}\left(2\right) & X_{32}\left(3\right) \end{array}\right],\;\mathrm{and}\\ \Phi & = & {\left[\begin{array}{cccccccc} \phi_{10}^{\left(1\right)} & \phi_{11}^{\left(1\right)} & \phi_{10}^{\left(2\right)} & \phi_{11}^{\left(2\right)} & \phi_{10}^{\left(3\right)} & \phi_{11}^{\left(3\right)} & \beta_{1} & \beta_{2} \end{array}\right]}^{⊤}. \end{array}$$

Straightforward modifications can be made to (6) and (8) for general p,N,T,m and d. Under regularity conditions, the authors of [40] have shown that the OLS estimator ${\hat{\Phi }}_{\mathrm{OLS}}$ is statistically consistent and follows the multivariate normal distribution asymptotically. However, as in seemingly unrelated regression models [41,42,43], when $\Sigma \neq \sigma^{2}I$, the OLS estimators are often not efficient [44]. The following estimator, derived in the spirit of generalised least squares (GLS) method is, in general, more efficient:

(9) $${\hat{\Phi }}_{\mathrm{GLS}}={\left(Y^{\prime }{\left(I⊗\Sigma \right)}^{-1}Y\right)}^{-1}Y^{⊤}{\left(I⊗\Sigma \right)}^{-1}Z,$$

where ⊗ denotes the Kronecker product [45].

However, given that Σ is rarely known, it has to be replaced by an estimate $\hat{\Sigma }$, yielding the feasible GLS estimator [46,47]:

(10) $${\hat{\Phi }}_{\mathrm{FGLS}}={\left(Y^{\prime }{\left(I⊗\hat{\Sigma }\right)}^{-1}Y\right)}^{-1}Y^{⊤}{\left(I⊗\hat{\Sigma }\right)}^{-1}Z.$$

A suitable estimate of Σ is

(11) $$\hat{\Sigma }=\frac{1}{T-p}\sum_{t=p+1}^{T}\hat{\varepsilon }\left(t\right)\hat{\varepsilon }{\left(t\right)}^{⊤},$$

where $\hat{\varepsilon }\left(t\right)={\left[{\hat{\varepsilon }}_{1}\left(t\right)\cdots {\hat{\varepsilon }}_{N}\left(t\right)\right]}^{⊤}$ represents the observed residuals. For small *p* and sufficiently large value of *T*, the denominator on the right hand side of (11) can be replaced by *T*. The standard errors of the estimates are the square root of the diagonal elements of the matrix ${\left(Y^{\prime }{\left(I⊗\hat{\Sigma }\right)}^{-1}Y\right)}^{-1}$.

### 3.2. Model Specifications

Some components in the GSTARX model have to be specified in advance. In our application, we had N=8,T=252 and m=9. The autoregressive order *p* was allowed to vary and the *p* which optimised some criteria described in Section 3.4 was selected. For the spatial orders, we fixed $\lambda_{1}=\lambda_{2}=\cdots =\lambda_{p}=1$. We focused on the first-order spatial effect for two reasons: (a) allowing $\lambda_{i}$s to be greater than one and vary among autoregressive orders would potentially lead to a large number of parameters, which may in turn lead to overfitting problems [48], and (b) the choice of temporal order is found to be more important than the spatial one, as the involvement of higher spatial orders does not guarantee substantial improvements in the goodness-of-fit [40]. In other words, only one spatial weight matrix W had to be specified. The following section details the requirements for a spatial weight matrix to be valid and the weight matrices used in our application.

As aforementioned, when some policies were not implemented in some of the locations, the GSTARX model under representation (2) cannot be used. In our application, for example, Level 3 border control had never taken place in ACT, NSW and VIC throughout the period of study. Thus, we resorted to use GSTARX model under representation (3), meaning that the effect of an intervention was assumed to be the same across all states and territories.

### 3.3. Spatial Weight Matrix

The spatial weight matrices are defined by the modeller. The following restrictions are imposed on $W^{\left(\ell \right)}$ to ensure the parameters are identifiable:

(a) $W^{\left(0\right)}=I_{N}$, the identity matrix of size *N*;
(b) for ℓ≥1, the weights $w_{ij}^{\left(\ell \right)}$ are non-zero only when locations *i* and *j* are *ℓ*-th order neighbours, and $w_{ii}^{\left(\ell \right)}=0$ for all *i* as a site is not a neighbour of itself by definition; and
(c) the weights are normalised in the sense that the sum of weights in each row of $W^{\left(\ell \right)}$ is 1, i.e., $\sum_{j=1}^{N}w_{ij}^{\left(\ell \right)}=1$.

**Figure 3:**
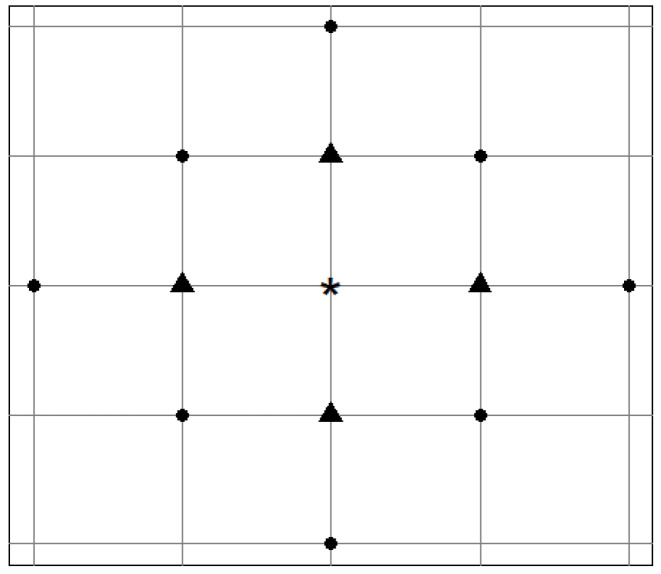
First- (triangles) and second- (circles) order spatial neighbours of a site (labelled by a star) in a regular grid.

By far, the most commonly used approaches rely on matrices with either the uniform weights [19] or the weights depending on the distances between the neighbouring sites [49]. Specifically, under the uniform weighting scheme, $w_{ij}^{\left(\ell \right)}=1/n_{ij}^{\left(\ell \right)}$ where $n_{ij}^{\left(\ell \right)}$ is the number of *ℓ*-th order neighbours for location *i*. In most applications, the spatial orders of 1 are used (that is, $\lambda_{1}=\lambda_{2}=\cdots =\lambda_{p}=1$) as the definitions of higher order spatial neighbours (and thus the weight matrices for ℓ>1) are not straightforward, except when the area of study is a regular grid [40]. In case the locations under consideration form a regular grid, the “first-order” neighbours can be defined as the sites that are immediately next to each other. The “second-order” neighbours can be defined as the first order neighbours of the first order neighbours. Figure 3 demonstrates the first- and second-order neighbours of a site based on such definition. For irregularly spaced areas, two areas can be considered as first-order neighbours if they share a border and second-order neighbours if they do not share a border but are separated by a common area.

In our application, the states and territories do not form a regular grid. The following spatial weight matrices were constructed and were tested:

$$W_{U}=\begin{array}{cc} \; & \begin{array}{cccccccc} ACT & \;NSW & \;NT & \;QLD & \;SA & \;TAS & \;VIC & \;WA \end{array}\\ \begin{array}{c} ACT\\ NSW\\ NT\\ QLD\\ SA\\ TAS\\ VIC\\ WA \end{array}\; & \;\left[\begin{array}{cccccccc} \;0\; & \;\;1/7\; & \;1/7\; & \;1/7\; & \;1/7\; & \;1/7\; & \;1/7\; & \;1/7\\ \;1/7\; & \;0\; & \;1/7\; & \;1/7\; & \;1/7\; & \;1/7\; & \;1/7\; & \;1/7\\ \;1/7\; & \;1/7\; & \;0\; & \;1/7\; & \;1/7\; & \;1/7\; & \;1/7\; & \;1/7\\ \;1/7\; & \;1/7\; & \;1/7\; & \;0\; & \;1/7\; & \;1/7\; & \;1/7\; & \;1/7\\ \;1/7\; & \;1/7\; & \;1/7\; & \;1/7\; & \;0\; & \;1/7\; & \;1/7\; & \;1/7\\ \;1/7\; & \;1/7\; & \;1/7\; & \;1/7\; & \;1/7\; & \;0\; & \;1/7\; & \;1/7\\ \;1/7\; & \;1/7\; & \;1/7\; & \;1/7\; & \;1/7\; & \;1/7\; & \;0\; & \;1/7\\ \;1/7\; & \;1/7\; & \;1/7\; & \;1/7\; & \;1/7\; & \;1/7\; & \;1/7\; & \;0 \end{array}\right] \end{array},$$

$$W_{B}=\begin{array}{cc} \; & \begin{array}{cccccccc} ACT & \;NSW & \;NT & \;QLD & \;SA & \;TAS & \;VIC & \;WA \end{array}\\ \begin{array}{c} ACT\\ NSW\\ NT\\ QLD\\ SA\\ TAS\\ VIC\\ WA \end{array}\; & \;\left[\begin{array}{cccccccc} 0\; & \;1\; & \;0\; & \;0\; & \;0\; & \;0\; & \;0\; & \;0\\ 1/4\; & \;0\; & \;0\; & \;1/4\; & \;1/4\; & \;0\; & \;1/4\; & \;0\\ 0\; & \;0\; & \;0\; & \;1/3\; & \;1/3\; & \;0\; & \;0\; & \;1/3\\ 0\; & \;1/3\; & \;1/3\; & \;0\; & \;1/3\; & \;0\; & \;0\; & \;0\\ 0\; & \;1/5\; & \;1/5\; & \;1/5\; & \;0\; & \;0\; & \;1/5\; & \;1/5\\ 0\; & \;0\; & \;0\; & \;0\; & \;0\; & \;0\; & \;1\; & \;0\\ 0\; & \;1/3\; & \;0\; & \;0\; & \;1/3\; & \;1/3\; & \;0\; & \;0\\ 0\; & \;0\; & \;1/2\; & \;0\; & \;1/2\; & \;0\; & \;0\; & \;0 \end{array}\right] \end{array},\;\mathrm{and}$$

$$W_{A}=\begin{array}{cc} \; & \begin{array}{cccccccc} ACT & \;NSW & \;NT & \;QLD & \;SA & \;TAS & \;VIC & \;WA \end{array}\\ \begin{array}{c} ACT\\ NSW\\ NT\\ QLD\\ SA\\ TAS\\ VIC\\ WA \end{array}\; & \;\left[\begin{array}{cccccccc} 0.000 & 0.279 & 0.000 & 0.256 & 0.062 & 0.000 & 0.402 & 0.000\\ 0.029 & 0.000 & 0.014 & 0.411 & 0.071 & 0.044 & 0.362 & 0.070\\ 0.000 & 0.291 & 0.000 & 0.299 & 0.088 & 0.000 & 0.201 & 0.120\\ 0.033 & 0.510 & 0.018 & 0.000 & 0.059 & 0.013 & 0.318 & 0.049\\ 0.026 & 0.283 & 0.017 & 0.191 & 0.000 & 0.000 & 0.380 & 0.103\\ 0.000 & 0.270 & 0.000 & 0.065 & 0.000 & 0.000 & 0.665 & 0.000\\ 0.043 & 0.378 & 0.010 & 0.268 & 0.099 & 0.112 & 0.000 & 0.090\\ 0.000 & 0.308 & 0.025 & 0.174 & 0.113 & 0.000 & 0.381 & 0.000 \end{array}\right] \end{array}.$$

The weight matrix $W_{U}$ was based on the uniform weighing scheme, assuming all states and territories were neighbours of each other. The weight matrix $W_{B}$ was based on the borders (see Figure 1). Two states are considered to be neighbours if they share a common border, with exception applied to Tasmania which does not share any borders with another state or territory. To satisfy the restriction that the sum of each row must be one, Tasmania and Victoria were considered neighbours. Finally, $W_{A}$ was based on domestic air travel activities. Specifically, we totalled the number of fare-paying passengers who travelled from one state or territory to another during the period from January to September 2020 by domestic flights listed in “top routes” from the statistical report released on 18 November 2020 by the Department of Infrastructure, Transport, Regional Development and Communications [50]. Compared to $W_{B}$, $W_{A}$ allows states and territories such as ACT to have more neighbours, as ACT is entirely surrounded by NSW. On the other hand, although $W_{U}$ was perhaps the most simplistic one, it allows all states and territories to interact with each other. Averages of these three matrices were also considered, namely, $W_{AB}=\left(W_{A}+W_{B}\right)/2$, $W_{AU}=\left(W_{A}+W_{U}\right)/2$, $W_{BU}=\left(W_{B}+W_{U}\right)/2$ and $W_{ABU}=\left(W_{A}+W_{B}+W_{U}\right)/3$.

### 3.4. Estimation

To estimate the autoregressive order *p* and the potential delay effects for the exogenous variables, a grid search was conducted. Specifically, we searched *p* between 1 and 14 (equivalent to a time frame between one day and two weeks) and $d=d_{0}$ between 0 and 7. During the grid search process, we fixed all $d_{i}=d_{0}$ for all i=1,2,…,m. For each combination of possible values of *p*, d and weight matrices, parameter estimation was carried out using the feasible GLS approach given in (10). Akaike information criterion (AIC [51]) was used to select the best combination of *p*, $d_{0}$ and W. For close AIC values, mean squared errors (MSE) were also considered. For each model, there were (2Np+m) parameters. Therefore, the selection criteria can be written as

$$\begin{array}{ccc} \mathrm{AIC} & = & -2\log\left(\hat{L}\right)+2\left(2Np+m\right)\\ \mathrm{MSE} & = & \frac{1}{N(T-p)}\sum_{i=1}^{N}\sum_{t=p+1}^{T}{\left[Z_{i}\left(t\right)-{\hat{Z}}_{i}\left(t\right)\right]}^{2}, \end{array}$$

where

$$\log\left(\hat{L}\right)=\sum_{t=p+1}^{T}\left[-\frac{N}{2}\log\left(2\pi \right)-\frac{1}{2}\log\left|\hat{\Sigma }\right|-\frac{1}{2}{\left[Z\left(t\right)-\hat{Z}\left(t\right)\right]}^{⊤}{\hat{\Sigma }}^{-1}\left[Z\left(t\right)-\hat{Z}\left(t\right)\right]\right]$$

is the observed log-likelihood value and ${\hat{Z}}_{i}\left(t\right)$ is the predicted value of $Z_{i}\left(t\right)$.

Once the optimal set of *p*, $d_{0}$ and W was decided, we conducted another round of grid search for $d_{i}$s within the interval of $d_{0}\pm 1$, assuming interventions from the same category had the same period of delay. That is, the periods of delay were assumed the same for $G_{1},G_{2}$ and $G_{3}$, and likewise for both *B* and *E*.

### 3.5. Statistical Software

All estimations were carried out using R [52]. The code used to produce the results is provided in Online Supplementary File S1. To use the code, one has to supply a T×N data matrix Z, a N×N spatial weight matrix W, a T×(N×m) covariate matrix X structured as $\left[X_{1},\cdots ,X_{m}\right]$, the number of covariates *m*, the autoregressive order *p*, the periods of delay d, a logical value TRUE or FALSE indicating if X has the same effect across all sites and the estimation method (either OLS or GLS). A GSTARX model can be fitted using the function GSTARX. A sample code with N=3, T=10, p=2, m=2, d=(1,2) and some randomly generated Z and X is provided at the bottom of the R code provided in Online Supplementary File S1.

## 4. Results

**Figure 4:**
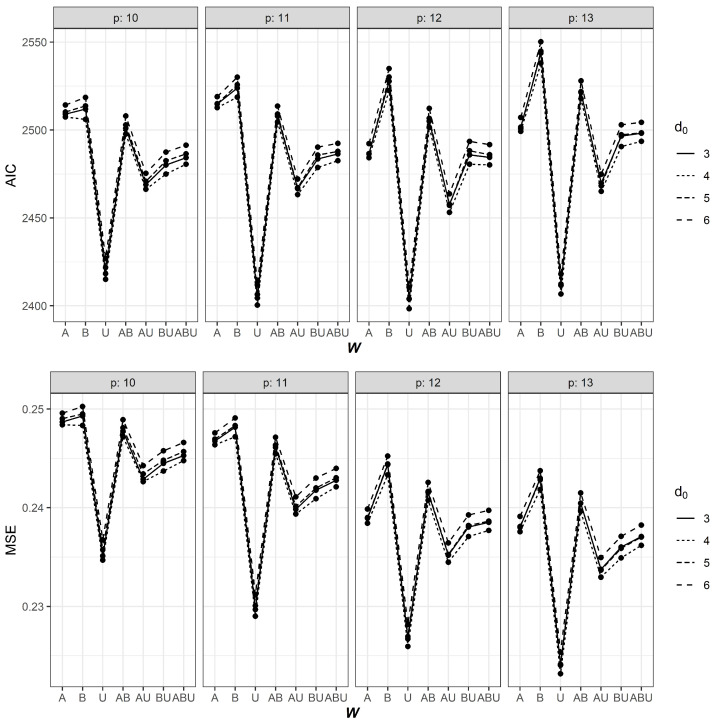
Values of AIC (top panel) and MSE (bottom panel) against different weight matrices *W* (horizontal axis) for $d_{0}=3,4,5,6$ and p=10,11,12,13.

The AIC and MSE values for some selected combinations of *p* and $d_{0}$ using different weight matrices are shown in Table 2. Results under all combinations of *p* and $d_{0}$ can be found in Tables S1–S9 in Online Supplementary File S2. A graphical representation is provided in Figure 4. The effect of $d_{0}$ on AIC and MSE was comparatively smaller than *p*. Nevertheless, the most influential element appeared to be the choice of the spatial weight matrix. It can be seen that the use of $W_{U}$ consistently provided the lowest AIC values. From Table 2 and Figure 4, it can be observed that the AIC and MSE values of using $W_{AU},W_{BU}$ and $W_{ABU}$ are, in general, lower than that of using $W_{A}$, $W_{B}$ and $W_{AB}$ but larger than that of using $W_{U}$. These results suggest that incorporating border or air travel information did not provide better fits, compared to a simpler uniform weighting scheme, for our data.

**Figure 5:**
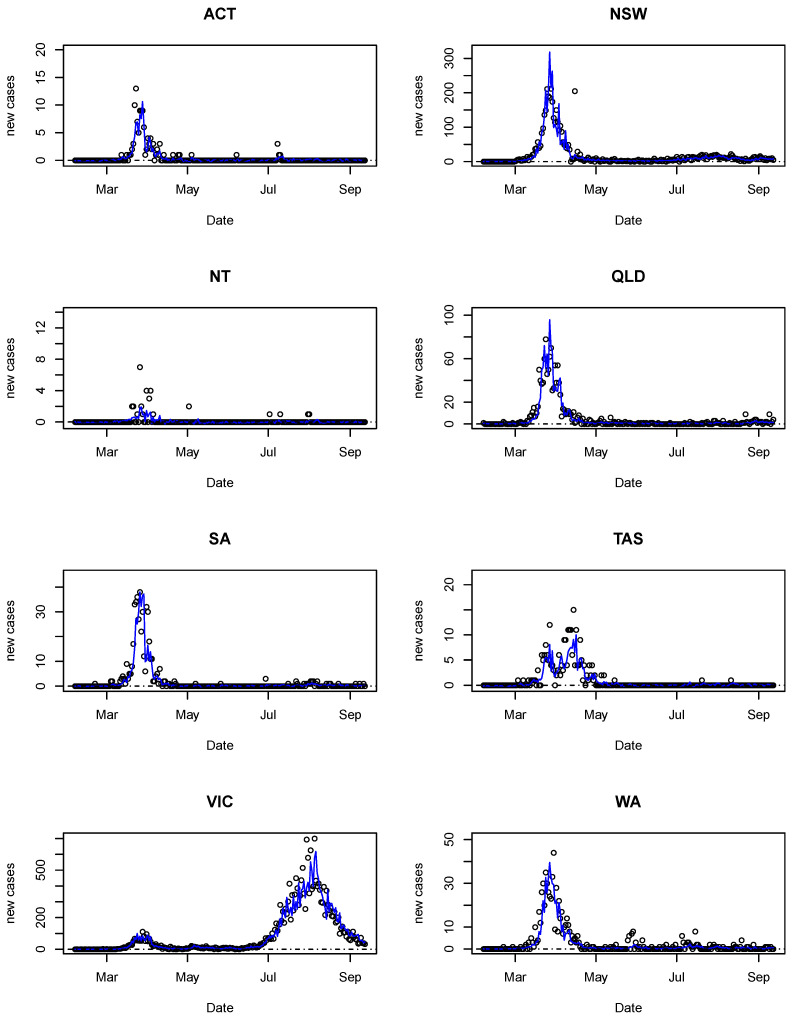
Observed (circle) and predicted (solid line) number of daily new cases in all states and territories. All the numbers have been transformed back to the original scale.

From Table 2, it can be observed that autoregressive orders 9, 11, 12 and 13 appear to be the most suitable for the dataset considered. The lowest AIC value was achieved when p=12 and $d_{0}=4$. The MSE of this model was only beaten by models with more parameters (that is, p=13), which is naturally expected as MSE tends to be lower when more parameters are involved. Overall, it seems a balance between goodness-of-fit and parsimony was attained using $d_{0}=4,p=12$ and $W=W_{U}$. Table 3 shows the AIC and MSE values when the periods of delay for *G*, *E* and *B* were varied between 3 and 5 days. It was found that a delay of 4 days for both *G* and *E* and a delay of 5 days for *B* yielded a slightly better fit (AIC = 2397.40, MSE = 0.226). Thus, we chose GSTARX(12;1;(4,4,4,4,4,4,5,5,5)) as the final model. The estimated parameters can be found in Table 4, while the fitted models are demonstrated in Figure 5.

Clear spatial variations can be seen in Figure 5. This figure also depicts that the fitted values matched well with the observed ones. The model was also able to capture the second wave that hit Victoria (mainly within Melbourne) in July 2020, which signifies the usefulness of the proposed method. Furthermore, it is interesting to observe that although state-level implementation of policies may vary, the model has produced fairly consistent results for all states except Tasmania, which showed somewhat a double peak between April and May 2020.

Findings from Table 4 reveal that the number of new cases depended on the number of cases in previous days recorded both in the same state or territory as well as in others. The dependence can be traced back to as far as 12 days. Such a result agrees well with other results from the literature. For example, the authors of [53] reported that the latency period of COVID-19 ranges from 1 to 14 days, while those of [54] reported a maximum median incubation period of 12 days. In addition, the World Health Organization also uses the history of travel or residence during the 14-day period prior to symptom onset to define a person as a suspect case [55].

Regarding the interventions, both economic and border restrictions tended to lower the number of new cases a couple of days after the implementations of the policies. Specifically, Level 3 economic restrictions (mandatory closure of non-essential businesses) reduced the number of cases by 0.15 standard deviations of the transformed variable (the actual numbers vary state by state). International border control ($B_{1}$) provided a similar effect, while interstate border control also helped in reducing the number of cases by approximately 0.13 standard deviations of the transformed variable.

On another note, the gathering restrictions, even the strictest level, resulted in positive changes in the number of cases. While this may seem surprising in the first instance, it is not illogical. First, although public gatherings were restricted, many people still went to school or work, especially essential workers. Cancelling public gathering alone did not fully stop social interactions. For example, people may still need to commute using public transportation such as trains, which is considered as having a high transmission risk [56]. Second, compared to economic and border restrictions, gathering restrictions are more challenging to enforce and rely on the awareness and discipline of the citizens.

## 5. Conclusions

To summarise, we have applied the GSTARX model with multiple interventions and delay effects incorporated to evaluate the effectiveness of COVID-19 mitigation policies implemented by Australian governments. The results showed that spatial and temporal dependencies existed for the number of daily new cases. Economic and border restrictions were found helpful in reducing the number of new cases a couple of days after implementation. It is worth emphasising that by no means all policies implemented were considered in our study. Some of the effects attributed to a particular intervention in this study may be merely due to some omitted variables.

A limitation of our approach lies in the use of a single weight matrix throughout the whole period of study. As a result of border restrictions, the neighbourhoods of each state or territory may change. In this regard, a time-varying model (see, e.g., in [57]) may be more suitable. However, such an approach requires extensive theoretical development, and we leave it as a direction for future research. In addition, note that the GSTARX model is purposely built for continuous data. Given the discrete nature of the data (counts), transformations were applied in the current work to achieve approximate normality. Developing models under the GSTARX framework that are specifically built for non-normal data can be considered as another direction for future research. Finally, given that Victoria has suffered from the second wave of COVID-19 outbreaks, our future research may also focus on assessing the robustness of mitigation strategies by comparing the responses of state and territory governments.

## Figures and Tables

**Table 1:** Descriptions of the policies considered in the analysis.

Policy	Description
Gathering	
Level 0	No upper limit on the number of people allowed.
Level 1 (Soft)	The upper limit was between 51 and 500.
Level 2 (Moderate)	The upper limit was between 3 and 50.
Level 3 (Strict)	The upper limit was 2.
Economy	
Level 0	No restrictions were imposed.
Level 1 (Soft)	Sit-down dining (with varying upper limit) at cafes, restaurants, pubs and clubs was allowed. Indoor religious gatherings (with varying upper limit) were allowed. Most indoor facilities such as gyms, libraries, museums were allowed to operate as long as some kind of “COVID Safe Plan” was enforced.
Level 2 (Moderate)	Access to some non-essential and leisure services allowed. Examples include outdoor, non-contact activities such as training and pools (indoor and outdoor), public spaces and lagoons, libraries, parks, playground equipment, skate parks and outdoor gyms. Recreational travel (possibly within certain distance from the place of residence) may be allowed.
Level 3 (Strict)	Mandatory closure of all non-essential services. Closure of places of social gathering, including registered and licensed clubs, licensed premises in hotels and bars and entertainment venues. Cafes and restaurants remain open but limited to only takeaway food. “Non-essential” businesses or activities including cinemas, casinos, concerts, indoor sports, gyms, playgrounds, campgrounds, libraries must not be operated.
Border Control	
Level 0	No restriction or international travel ban imposed on certain countries.
Level 1	Closure of international border. No interstate border control was in place.
Level 2	Closure of international border. Interstate border control was applied to one state or territory.
Level 3	Closure of international border. Interstate border control was applied to multiple states and territories.

**Table 2:** Selected list of AIC and MSE values under various combinations of *p* and $d_{0}$ using different weight matrices. For each value of $d_{0}$, the values of *p* that resulted in the lowest three AIC values under $W_{U}$ are provided. The overall lowest three values of AIC were indicated by boldface. Full results are provided in Online Supplementary File S2.

		$W_{A}$		$W_{B}$		$W_{U}$		$W_{\mathrm{AB}}$		$W_{\mathrm{AU}}$		$W_{\mathrm{BU}}$		$W_{\mathrm{ABU}}$	
$d_{0}$	p	**AIC**	**MSE**	**AIC**	**MSE**	**AIC**	**MSE**	**AIC**	**MSE**	**AIC**	**MSE**	**AIC**	**MSE**	**AIC**	**MSE**
0	11	2530.2	0.249	2533.7	0.248	2416.2	0.231	2522.5	0.248	2479.0	0.242	2494.4	0.243	2499.6	0.245
	12	2500.1	0.241	2536.6	0.245	2414.5	0.229	2519.0	0.243	2469.2	0.237	2496.5	0.239	2497.4	0.240
	13	2514.9	0.240	2552.1	0.243	2424.2	0.226	2535.1	0.242	2481.8	0.236	2507.2	0.237	2511.2	0.239
1	9	2513.3	0.253	2514.0	0.251	2418.4	0.238	2508.0	0.251	2469.8	0.247	2478.8	0.247	2486.8	0.249
	11	2523.1	0.250	2526.5	0.250	2410.7	0.231	2514.7	0.248	2474.6	0.242	2488.6	0.243	2493.6	0.244
	12	2494.0	0.241	2530.2	0.245	2410.0	0.228	2511.9	0.243	2465.4	0.237	2491.1	0.239	2491.8	0.240
2	11	2518.0	0.248	2527.3	0.249	2407.1	0.230	2511.9	0.247	2469.3	0.240	2486.6	0.242	2489.9	0.244
	12	2490.5	0.240	2530.9	0.245	2405.8	0.227	2509.6	0.242	2460.6	0.236	2489.0	0.239	2488.5	0.239
	13	2505.6	0.239	2546.3	0.243	2414.8	0.225	2525.7	0.241	2473.1	0.234	2499.4	0.237	2502.1	0.238
3	9	2504.1	0.251	2510.1	0.251	2411.2	0.236	2499.6	0.246	2461.0	0.240	2472.6	0.242	2478.3	0.243
	11	2509.0	0.249	2511.9	0.249	2404.4	0.230	2507.9	0.242	2466.6	0.235	2483.5	0.238	2486.4	0.239
	12	2486.5	0.239	2527.7	0.244	**2403.9**	0.227	2505.0	0.240	2457.7	0.234	2485.9	0.236	2484.4	0.237
4	11	2512.7	0.246	2518.7	0.247	**2400.4**	0.229	2504.6	0.245	2463.3	0.239	2478.7	0.241	2482.6	0.242
	12	2484.2	0.238	2522.5	0.243	**2398.3**	0.226	2501.6	0.241	2453.2	0.234	2480.6	0.237	2480.1	0.238
	13	2499.2	0.238	2538.0	0.242	2406.7	0.223	2517.8	0.240	2465.1	0.233	2490.6	0.235	2493.5	0.236
5	11	2515.0	0.247	2525.8	0.248	2406.4	0.230	2509.1	0.246	2467.2	0.240	2485.7	0.242	2487.8	0.243
	12	2486.4	0.239	2530.1	0.244	2404.0	0.227	2506.6	0.242	2457.2	0.235	2488.2	0.238	2486.0	0.239
	13	2500.7	0.238	2544.8	0.243	2411.6	0.224	2521.8	0.240	2468.3	0.234	2497.3	0.236	2498.4	0.237
6	11	2518.9	0.248	2530.1	0.249	2411.9	0.231	2513.6	0.247	2472.1	0.241	2490.3	0.243	2492.4	0.244
	12	2492.2	0.240	2534.9	0.245	2410.5	0.228	2512.3	0.243	2463.7	0.236	2493.5	0.239	2491.7	0.240
	13	2507.1	0.239	2550.2	0.244	2418.0	0.225	2528.0	0.241	2474.8	0.235	2502.9	0.237	2504.4	0.238
7	11	2524.2	0.248	2537.5	0.249	2413.5	0.231	2520.0	0.248	2475.0	0.241	2495.2	0.243	2497.2	0.244
	12	2497.7	0.240	2541.1	0.245	2410.6	0.228	2517.7	0.243	2465.2	0.236	2497.5	0.239	2495.3	0.240
	13	2511.7	0.239	2555.0	0.243	2418.3	0.225	2532.5	0.242	2476.6	0.235	2506.6	0.237	2507.8	0.238

**Table 3:** AIC and MSE values for different combinations of $d_{G}$, $d_{E}$ and $d_{B}$ (periods of delay for *G*, *E* and *B*, respectively) within $d_{0}\pm 1$. Lowest values of AIC and MSE are indicated by boldface.

	**AIC**								
	$d_{G}=3$			$d_{G}=4$			$d_{G}=5$		
	$d_{E}=3$	$d_{E}=4$	$d_{E}=5$	$d_{E}=3$	$d_{E}=4$	$d_{E}=5$	$d_{E}=3$	$d_{E}=4$	$d_{E}=5$
$d_{B}=3$	2403.9	2401.3	2401.5	2402.4	2399.5	2400.3	2408.2	2406.0	2406.2
$d_{B}=4$	2402.8	2400.4	2400.6	2400.9	2398.3	2398.9	2406.8	2405.0	2405.2
$d_{B}=5$	2401.8	2399.9	2399.7	2399.5	**2397.4**	2397.7	2405.3	2404.0	2404.0
	**MSE**								
	$d_{G}=3$			$d_{G}=4$			$d_{G}=5$		
	$d_{E}=3$	$d_{E}=4$	$d_{E}=5$	$d_{E}=3$	$d_{E}=4$	$d_{E}=5$	$d_{E}=3$	$d_{E}=4$	$d_{E}=5$
$d_{B}=3$	0.2267	0.2263	0.2263	0.2265	0.2259	0.2260	0.2273	0.2270	0.2270
$d_{B}=4$	0.2267	0.2264	0.2264	0.2265	0.2259	0.2260	0.2274	0.2271	0.2271
$d_{B}=5$	0.2265	0.2262	0.2263	0.2262	**0.2258**	0.2258	0.2271	0.2269	0.2269

**Table 4:** Feasible GLS estimates of parameters and standard errors (in parentheses) of the fitted GSTARX(12;1;(4,4,4,4,4,4,5,5,5)). *p*-values less than 0.01, 0.05 and 0.1 are indicated by three, two and one asterisk(s), respectively.

$\phi_{k0}$								
k	**ACT**	**NSW**	**NT**	**QLD**	**SA**	**TAS**	**VIC**	**WA**
1	0.364 ***	0.317 ***	0.099	0.228 ***	0.3 ***	0.141 **	0.425 ***	0.201 ***
	(0.064)	(0.067)	(0.066)	(0.065)	(0.067)	(0.066)	(0.066)	(0.066)
2	0.138 **	0.085	−0.072	0.166 **	0.032	0.353 ***	0.06	0.132 *
	(0.068)	(0.071)	(0.066)	(0.067)	(0.07)	(0.066)	(0.072)	(0.067)
3	0.008	0.035	0.075	0.051	0.123 *	0.146 **	0.396 ***	0.159 **
	(0.068)	(0.071)	(0.067)	(0.07)	(0.069)	(0.07)	(0.072)	(0.068)
4	0.135 **	0.156 **	−0.221 ***	0.072	0.004	0.034	0.052	0.006
	(0.068)	(0.07)	(0.064)	(0.071)	(0.067)	(0.07)	(0.076)	(0.069)
5	0.096	0.158 **	0.174 ***	0.138 *	−0.067	−0.021	0.187 **	0.141 **
	(0.067)	(0.07)	(0.067)	(0.071)	(0.068)	(0.069)	(0.075)	(0.07)
6	−0.007	0.028	0.028	0.086	0.117 *	0.052	−0.055	0.047
	(0.067)	(0.072)	(0.066)	(0.07)	(0.067)	(0.069)	(0.076)	(0.07)
7	−0.077	−0.002	0.264 ***	0.12 *	0.092	−0.06	−0.037	−0.076
	(0.067)	(0.16)	(0.065)	(0.071)	(0.068)	(0.069)	(0.076)	(0.071)
8	−0.234 ***	0.173 **	−0.039	0.098	−0.025	0.041	−0.102	−0.057
	(0.067)	(0.073)	(0.067)	(0.07)	(0.069)	(0.069)	(0.075)	(0.071)
9	−0.038	−0.145 **	−0.116 *	0.011	0.068	0.056	−0.19 **	−0.042
	(0.065)	(0.072)	(0.066)	(0.071)	(0.069)	(0.068)	(0.074)	(0.071)
10	0.16 **	0.106	0.027	−0.088	0.07	−0.134 **	0.071	0.066
	(0.065)	(0.071)	(0.07)	(0.072)	(0.069)	(0.068)	(0.07)	(0.07)
11	−0.093	−0.014	−0.071	0.028	−0.092	0.051	0.031	−0.011
	(0.064)	(0.07)	(0.069)	(0.071)	(0.069)	(0.064)	(0.069)	(0.07)
12	0.135 **	−0.044	−0.1	−0.105	0.025	0.031	0.097	−0.013
	(0.06)	(0.067)	(0.069)	(0.069)	(0.065)	(0.06)	(0.064)	(0.068)
$\phi_{k1}$								
k	**ACT**	**NSW**	**NT**	**QLD**	**SA**	**TAS**	**VIC**	**WA**
1	0.345 ***	0.345 **	-0.114	0.508 ***	0.352 ***	0.573 ***	0.018	0.374 **
	(0.134)	(0.14)	(0.261)	(0.142)	(0.133)	(0.128)	(0.067)	(0.162)
2	0.449 ***	−0.253	0.434	−0.053	0.371 **	0.161	0.068	−0.09
	(0.152)	(0.162)	(0.315)	(0.159)	(0.149)	(0.152)	(0.077)	(0.185)
3	−0.081	0.336 **	−0.124	−0.011	−0.02	0.04	−0.204 ***	0.079
	(0.156)	(0.166)	(0.322)	(0.161)	(0.15)	(0.154)	(0.079)	(0.189)
4	−0.682 ***	−0.133	0.698 **	−0.09	−0.077	−0.621 ***	0.178 **	0.158
	(0.155)	(0.158)	(0.312)	(0.154)	(0.147)	(0.145)	(0.078)	(0.183)
5	0.323 **	0.038	−0.097	−0.06	0.329 **	0.102	0.036	−0.103
	(0.161)	(0.159)	(0.313)	(0.155)	(0.148)	(0.151)	(0.08)	(0.187)
6	0.216	0.038	0.194	−0.245	−0.049	−0.043	0.059	0.193
	(0.164)	(0.073)	(0.311)	(0.158)	(0.149)	(0.151)	(0.081)	(0.19)
7	0.28 *	0.169	−0.495	0.273 *	0.04	−0.123	−0.004	0.014
	(0.164)	(0.159)	(0.311)	(0.159)	(0.149)	(0.15)	(0.08)	(0.189)
8	0.175	−0.019	0.835 ***	−0.119	0.01	−0.306 **	0.107	0.16
	(0.163)	(0.155)	(0.308)	(0.156)	(0.146)	(0.147)	(0.079)	(0.186)
9	−0.256	−0.43 ***	−0.664 **	−0.062	−0.556 ***	0.085	−0.223 ***	−0.159
k	**ACT**	**NSW**	**NT**	**QLD**	**SA**	**TAS**	**VIC**	**WA**
	(0.163)	(0.154)	(0.307)	(0.154)	(0.144)	(0.148)	(0.078)	(0.181)
10	0.257	0.105	0.529 *	0.177	−0.227	0.094	0.12	0.203
	(0.163)	(0.164)	(0.306)	(0.159)	(0.15)	(0.154)	(0.081)	(0.186)
11	−0.315 *	−0.174	−0.541 *	0.132	0.303 **	0.406 ***	0.038	−0.259
	(0.162)	(0.161)	(0.306)	(0.156)	(0.15)	(0.153)	(0.08)	(0.184)
12	−0.366 **	0.069	−0.178	−0.358 ***	−0.179	0.061	−0.22 ***	−0.159
	(0.143)	(0.141)	(0.251)	(0.136)	(0.136)	(0.141)	(0.068)	(0.158)
β								
$G_{1}$	$G_{2}$	$G_{3}$	$E_{1}$	$E_{2}$	$E_{3}$	$B_{1}$	$B_{2}$	$B_{3}$
0.136 **	0.224 ***	0.344 ***	−0.089	−0.11	−0.151 *	−0.15 **	−0.129 *	−0.125 **
(0.056)	(0.056)	(0.067)	(0.07)	(0.074)	(0.084)	(0.059)	(0.074)	(0.049)

## Data Availability

The data used for this study are publicly available on the webpage of Department of Health, Australia Government: https://www.health.gov.au/news/health-alerts/novel-coronavirus-2019-ncov-health-alert/coronavirus-covid-19-current-situation-and-case-numbers. Last accessed: 18 November 2020.

## Supplementary Materials

The following are available online at https://www.mdpi.com/article/10.3390/ijerph18147474/s1, S1: R code for GLS estimation of GSTARX model, S2: Additional Estimation Results.
