# Supplementary material for: Evaluation of COVID-19 Mitigation Policies in Australia Using Generalised Space-Time Autoregressive Intervention Models
Source: Int J Environ Res Public Health. 2021 Jul 13;18(14):7474. doi: 10.3390/ijerph18147474 (PMC8306935; doi:10.3390/ijerph18147474)
Supplement: Supplementary file 1 [file ijerph-18-07474-s001.zip › OnlineSuppFile2_AddTables.pdf]

Evaluation of COVID-19 Mitigation Policies in Australia Using Generalised Space-Time Autoregressive Intervention Models

Online Supplementary File 2 – Additional Estimation Results

Ryan H.L. Ip*, Dmitry Demskoi, Azizur Rahman, Lihong Zheng
School of Computing and Mathematics, Charles Sturt University, Australia

*Email: hoip@csu.edu.au

	AIC							MSE						
p	$\mathbf{W}_A$	$\mathbf{W}_B$	$\mathbf{W}_U$	$\mathbf{W}_{AB}$	$\mathbf{W}_{AU}$	$\mathbf{W}_{BU}$	$\mathbf{W}_{ABU}$	$\mathbf{W}_A$	$\mathbf{W}_B$	$\mathbf{W}_U$	$\mathbf{W}_{AB}$	$\mathbf{W}_{AU}$	$\mathbf{W}_{BU}$	$\mathbf{W}_{ABU}$
1	2800.4	2799.8	2707.1	2779.8	2747.6	2754.2	2754.8	0.323	0.318	0.309	0.317	0.316	0.313	0.315
2	2636.7	2625.8	2584.7	2620.8	2612.7	2601.9	2608.8	0.294	0.289	0.286	0.290	0.291	0.287	0.289
3	2578.3	2574.2	2534.4	2571.0	2560.9	2557.2	2562.8	0.286	0.282	0.280	0.283	0.284	0.281	0.283
4	2578.0	2571.0	2509.3	2569.5	2546.5	2546.9	2554.6	0.280	0.276	0.270	0.277	0.276	0.273	0.275
5	2540.7	2557.4	2497.1	2548.8	2522.0	2536.4	2537.0	0.269	0.270	0.264	0.269	0.267	0.267	0.268
6	2537.9	2548.6	2486.1	2541.7	2516.8	2527.7	2530.3	0.267	0.267	0.260	0.267	0.265	0.264	0.265
7	2533.7	2551.3	2482.4	2541.1	2514.4	2528.1	2529.6	0.261	0.262	0.254	0.261	0.259	0.259	0.260
8	2546.3	2548.4	2479.0	2545.6	2520.5	2522.2	2531.3	0.260	0.259	0.252	0.259	0.257	0.256	0.258
9	2521.7	2521.1	2424.7	2516.6	2475.2	2485.1	2493.7	0.254	0.251	0.238	0.252	0.247	0.247	0.249
10	2527.2	2522.6	2430.6	2518.1	2483.3	2491.8	2499.4	0.251	0.249	0.237	0.250	0.245	0.245	0.247
11	2530.2	2533.7	2416.2	2522.5	2479.0	2494.4	2499.6	0.249	0.248	0.231	0.248	0.242	0.243	0.245
12	2500.1	2536.6	2414.5	2519.0	2469.2	2496.5	2497.4	0.241	0.245	0.229	0.243	0.237	0.239	0.240
13	2514.9	2552.1	2424.2	2535.1	2481.8	2507.2	2511.2	0.240	0.243	0.226	0.242	0.236	0.237	0.239
14	2526.4	2559.2	2432.4	2545.6	2495.5	2515.9	2522.7	0.239	0.242	0.224	0.241	0.235	0.236	0.238

Table S.1: AIC and MSE under different combinations of p and $\mathbf{W}$ for $d_0 = 0$.

	AIC							MSE						
p	$\mathbf{W}_A$	$\mathbf{W}_B$	$\mathbf{W}_U$	$\mathbf{W}_{AB}$	$\mathbf{W}_{AU}$	$\mathbf{W}_{BU}$	$\mathbf{W}_{ABU}$	$\mathbf{W}_A$	$\mathbf{W}_B$	$\mathbf{W}_U$	$\mathbf{W}_{AB}$	$\mathbf{W}_{AU}$	$\mathbf{W}_{BU}$	$\mathbf{W}_{ABU}$
1	2796.6	2800.6	2707.7	2778.8	2746.8	2756.1	2755.1	0.322	0.319	0.308	0.317	0.316	0.313	0.314
2	2629.9	2622.9	2581.1	2616.1	2608.4	2599.4	2605.1	0.293	0.289	0.286	0.289	0.291	0.287	0.289
3	2570.0	2569.4	2529.8	2564.3	2555.2	2552.7	2557.1	0.285	0.282	0.279	0.283	0.284	0.281	0.282
4	2569.5	2565.7	2504.2	2562.3	2540.4	2541.8	2548.4	0.279	0.276	0.270	0.277	0.275	0.273	0.275
5	2532.7	2551.0	2491.4	2541.3	2516.0	2530.3	2530.4	0.269	0.270	0.264	0.269	0.267	0.267	0.268
6	2528.7	2541.7	2479.9	2533.5	2510.0	2521.1	2523.1	0.267	0.267	0.260	0.267	0.265	0.264	0.265
7	2524.1	2543.5	2476.0	2531.8	2507.5	2520.9	2521.7	0.261	0.262	0.254	0.261	0.258	0.259	0.260
8	2536.8	2540.3	2471.4	2536.2	2513.2	2514.6	2523.1	0.260	0.259	0.251	0.259	0.257	0.256	0.258
9	2513.3	2514.0	2418.4	2508.0	2469.8	2478.8	2486.8	0.253	0.251	0.238	0.251	0.247	0.247	0.249
10	2518.2	2515.0	2424.3	2508.9	2477.2	2485.2	2491.9	0.250	0.250	0.236	0.249	0.245	0.245	0.247
11	2523.1	2526.5	2410.7	2514.7	2474.6	2488.6	2493.6	0.249	0.249	0.231	0.248	0.242	0.243	0.244
12	2494.0	2530.2	2410.0	2511.9	2465.4	2491.1	2491.8	0.241	0.245	0.228	0.243	0.237	0.239	0.240
13	2509.4	2546.5	2419.1	2528.7	2477.7	2501.9	2505.8	0.240	0.244	0.226	0.242	0.236	0.237	0.239
14	2522.4	2554.2	2427.4	2540.2	2492.2	2510.9	2517.9	0.239	0.242	0.223	0.241	0.235	0.236	0.238

Table S.2: AIC and MSE under different combinations of p and $\mathbf{W}$ for $d_0 = 1$.

	AIC							MSE						
p	$\mathbf{W}_A$	$\mathbf{W}_B$	$\mathbf{W}_U$	$\mathbf{W}_{AB}$	$\mathbf{W}_{AU}$	$\mathbf{W}_{BU}$	$\mathbf{W}_{ABU}$	$\mathbf{W}_A$	$\mathbf{W}_B$	$\mathbf{W}_U$	$\mathbf{W}_{AB}$	$\mathbf{W}_{AU}$	$\mathbf{W}_{BU}$	$\mathbf{W}_{ABU}$
1	2790.1	2797.6	2700.3	2773.5	2738.3	2750.8	2748.5	0.321	0.318	0.307	0.316	0.314	0.312	0.313
2	2625.2	2621.1	2575.5	2611.9	2602.1	2595.6	2600.0	0.293	0.289	0.285	0.289	0.290	0.286	0.288
3	2567.1	2568.9	2526.0	2561.6	2550.8	2550.6	2553.8	0.285	0.283	0.279	0.283	0.283	0.281	0.282
4	2566.7	2565.6	2500.8	2559.8	2536.6	2540.1	2545.4	0.279	0.277	0.269	0.277	0.275	0.273	0.275
5	2529.2	2551.0	2488.3	2538.9	2512.2	2529.0	2527.7	0.269	0.271	0.263	0.270	0.267	0.268	0.268
6	2525.8	2542.2	2477.4	2531.7	2506.8	2520.2	2521.0	0.267	0.268	0.260	0.267	0.264	0.265	0.265
7	2521.4	2544.8	2473.8	2530.6	2504.4	2520.5	2519.9	0.261	0.263	0.253	0.262	0.258	0.259	0.260
8	2533.7	2541.0	2469.1	2534.3	2509.7	2513.7	2520.7	0.259	0.260	0.251	0.259	0.256	0.256	0.257
9	2508.5	2514.4	2415.1	2505.1	2465.2	2477.2	2483.5	0.253	0.252	0.237	0.251	0.246	0.247	0.248
10	2513.5	2515.8	2421.8	2506.1	2472.8	2483.9	2488.8	0.250	0.250	0.236	0.249	0.244	0.245	0.246
11	2518.0	2527.3	2407.1	2511.9	2469.3	2486.6	2489.9	0.248	0.249	0.230	0.247	0.240	0.242	0.244
12	2490.5	2530.9	2405.8	2509.6	2460.6	2489.0	2488.5	0.240	0.245	0.227	0.242	0.236	0.239	0.239
13	2505.6	2546.3	2414.8	2525.7	2473.1	2499.4	2502.1	0.239	0.243	0.225	0.241	0.234	0.237	0.238
14	2517.8	2553.4	2422.5	2536.4	2486.6	2507.8	2513.4	0.238	0.242	0.222	0.240	0.233	0.235	0.237

Table S.3: AIC and MSE under different combinations of p and $\mathbf{W}$ for $d_0 = 2$.

	AIC							MSE						
p	$\mathbf{W}_A$	$\mathbf{W}_B$	$\mathbf{W}_U$	$\mathbf{W}_{AB}$	$\mathbf{W}_{AU}$	$\mathbf{W}_{BU}$	$\mathbf{W}_{ABU}$	$\mathbf{W}_A$	$\mathbf{W}_B$	$\mathbf{W}_U$	$\mathbf{W}_{AB}$	$\mathbf{W}_{AU}$	$\mathbf{W}_{BU}$	$\mathbf{W}_{ABU}$
1	2791.4	2799.1	2699.9	2774.7	2739.2	2751.8	2749.6	0.321	0.318	0.307	0.315	0.314	0.313	0.313
2	2623.4	2619.9	2571.2	2609.3	2598.4	2592.8	2596.7	0.292	0.289	0.285	0.288	0.289	0.286	0.287
3	2564.9	2567.8	2522.9	2558.7	2547.6	2548.1	2550.5	0.285	0.282	0.278	0.282	0.282	0.281	0.282
4	2564.3	2564.8	2497.5	2557.0	2533.3	2537.6	2542.1	0.278	0.276	0.269	0.276	0.274	0.273	0.274
5	2527.5	2551.2	2486.0	2537.0	2509.9	2527.2	2525.3	0.268	0.271	0.263	0.269	0.266	0.267	0.268
6	2522.9	2540.4	2474.3	2528.3	2503.7	2517.2	2517.4	0.266	0.268	0.259	0.266	0.264	0.264	0.265
7	2517.9	2543.2	2470.3	2527.0	2500.9	2517.4	2516.1	0.260	0.263	0.253	0.261	0.257	0.259	0.259
8	2530.2	2538.6	2465.1	2530.3	2506.0	2509.8	2516.4	0.258	0.259	0.250	0.258	0.255	0.256	0.256
9	2504.1	2510.1	2411.2	2499.6	2461.0	2472.6	2478.3	0.251	0.251	0.236	0.250	0.245	0.246	0.247
10	2509.0	2511.9	2418.3	2501.0	2468.9	2480.1	2484.2	0.249	0.249	0.235	0.248	0.243	0.244	0.245
11	2514.7	2523.9	2404.4	2507.9	2466.6	2483.5	2486.4	0.247	0.248	0.230	0.246	0.240	0.242	0.243
12	2486.5	2527.7	2403.9	2505.0	2457.7	2485.9	2484.4	0.239	0.244	0.227	0.242	0.235	0.238	0.239
13	2501.6	2543.7	2412.4	2521.4	2469.8	2496.6	2498.2	0.238	0.243	0.224	0.240	0.234	0.236	0.237
14	2514.0	2551.3	2420.4	2532.2	2483.6	2505.2	2509.7	0.237	0.242	0.221	0.240	0.232	0.234	0.236

Table S.4: AIC and MSE under different combinations of p and $\mathbf{W}$ for $d_0 = 3$.

	AIC							MSE						
p	$\mathbf{W}_A$	$\mathbf{W}_B$	$\mathbf{W}_U$	$\mathbf{W}_{AB}$	$\mathbf{W}_{AU}$	$\mathbf{W}_{BU}$	$\mathbf{W}_{ABU}$	$\mathbf{W}_A$	$\mathbf{W}_B$	$\mathbf{W}_U$	$\mathbf{W}_{AB}$	$\mathbf{W}_{AU}$	$\mathbf{W}_{BU}$	$\mathbf{W}_{ABU}$
1	2788.8	2795.9	2696.6	2772.0	2736.6	2748.4	2746.7	0.321	0.318	0.307	0.316	0.314	0.312	0.313
2	2621.0	2616.5	2567.6	2606.4	2595.4	2589.1	2593.5	0.292	0.288	0.284	0.288	0.289	0.285	0.287
3	2564.1	2565.6	2520.3	2557.1	2545.4	2545.3	2548.2	0.284	0.281	0.278	0.281	0.282	0.280	0.281
4	2562.7	2561.3	2495.2	2554.2	2530.8	2533.7	2538.9	0.278	0.275	0.268	0.275	0.274	0.272	0.273
5	2525.5	2546.3	2483.0	2533.4	2506.8	2522.4	2521.3	0.268	0.270	0.262	0.268	0.266	0.266	0.267
6	2521.6	2535.3	2472.5	2525.0	2501.6	2512.8	2514.1	0.266	0.267	0.259	0.266	0.263	0.263	0.264
7	2515.4	2537.3	2468.1	2522.9	2498.1	2512.5	2512.2	0.259	0.262	0.252	0.260	0.257	0.258	0.258
8	2528.7	2534.0	2464.3	2527.7	2504.6	2506.5	2514.0	0.258	0.258	0.250	0.258	0.255	0.255	0.256
9	2502.8	2504.1	2407.6	2496.5	2458.5	2467.5	2474.9	0.251	0.250	0.236	0.249	0.244	0.245	0.246
10	2507.4	2506.0	2415.1	2497.7	2466.3	2474.9	2480.6	0.248	0.248	0.235	0.247	0.243	0.244	0.245
11	2512.7	2518.7	2400.4	2504.6	2463.3	2478.7	2482.6	0.246	0.247	0.229	0.245	0.239	0.241	0.242
12	2484.2	2522.5	2398.3	2501.6	2453.2	2480.6	2480.1	0.238	0.243	0.226	0.241	0.234	0.237	0.238
13	2499.2	2538.0	2406.7	2517.8	2465.1	2490.6	2493.5	0.238	0.242	0.223	0.240	0.233	0.235	0.236
14	2512.6	2547.0	2415.3	2530.0	2479.5	2500.5	2506.1	0.237	0.241	0.221	0.239	0.232	0.233	0.235

Table S.5: AIC and MSE under different combinations of p and $\mathbf{W}$ for $d_0 = 4$.

	AIC							MSE						
p	$\mathbf{W}_A$	$\mathbf{W}_B$	$\mathbf{W}_U$	$\mathbf{W}_{AB}$	$\mathbf{W}_{AU}$	$\mathbf{W}_{BU}$	$\mathbf{W}_{ABU}$	$\mathbf{W}_A$	$\mathbf{W}_B$	$\mathbf{W}_U$	$\mathbf{W}_{AB}$	$\mathbf{W}_{AU}$	$\mathbf{W}_{BU}$	$\mathbf{W}_{ABU}$
1	2788.6	2798.0	2698.6	2773.2	2737.2	2750.3	2748.0	0.321	0.318	0.307	0.316	0.314	0.313	0.313
2	2622.9	2620.3	2572.7	2609.8	2598.8	2593.6	2597.3	0.293	0.289	0.285	0.289	0.290	0.286	0.288
3	2567.6	2570.4	2525.5	2561.6	2549.5	2550.8	2553.0	0.285	0.282	0.279	0.282	0.283	0.280	0.281
4	2565.9	2566.8	2500.3	2558.9	2534.7	2539.6	2543.9	0.279	0.276	0.269	0.276	0.274	0.273	0.274
5	2528.9	2552.8	2487.6	2538.6	2510.8	2528.7	2526.6	0.268	0.270	0.263	0.269	0.266	0.267	0.267
6	2525.7	2543.5	2477.3	2531.1	2506.0	2520.0	2520.0	0.266	0.267	0.260	0.266	0.264	0.264	0.265
7	2520.1	2546.0	2474.2	2529.4	2503.3	2520.5	2518.7	0.260	0.263	0.253	0.261	0.258	0.259	0.259
8	2532.3	2541.3	2469.6	2532.7	2508.8	2513.3	2519.3	0.259	0.259	0.251	0.258	0.256	0.256	0.257
9	2506.1	2511.7	2415.0	2501.9	2463.4	2475.2	2480.8	0.252	0.251	0.237	0.250	0.245	0.246	0.247
10	2510.3	2513.7	2421.8	2502.9	2470.8	2482.5	2486.4	0.249	0.249	0.236	0.248	0.243	0.245	0.246
11	2515.0	2525.8	2406.4	2509.1	2467.2	2485.7	2487.8	0.247	0.248	0.230	0.246	0.240	0.242	0.243
12	2486.4	2530.1	2404.0	2506.6	2457.2	2488.2	2486.0	0.239	0.244	0.227	0.242	0.235	0.238	0.239
13	2500.7	2544.8	2411.6	2521.8	2468.3	2497.3	2498.4	0.238	0.243	0.224	0.240	0.234	0.236	0.237
14	2513.9	2553.0	2419.5	2533.8	2482.1	2506.6	2510.5	0.237	0.242	0.221	0.240	0.232	0.234	0.236

Table S.6: AIC and MSE under different combinations of p and $\mathbf{W}$ for $d_0 = 5$.

	AIC							MSE						
p	$\mathbf{W}_A$	$\mathbf{W}_B$	$\mathbf{W}_U$	$\mathbf{W}_{AB}$	$\mathbf{W}_{AU}$	$\mathbf{W}_{BU}$	$\mathbf{W}_{ABU}$	$\mathbf{W}_A$	$\mathbf{W}_B$	$\mathbf{W}_U$	$\mathbf{W}_{AB}$	$\mathbf{W}_{AU}$	$\mathbf{W}_{BU}$	$\mathbf{W}_{ABU}$
1	2792.7	2802.0	2706.2	2778.4	2743.9	2756.2	2754.2	0.322	0.319	0.309	0.317	0.316	0.314	0.315
2	2625.3	2623.5	2579.8	2613.6	2603.8	2598.6	2602.1	0.293	0.289	0.286	0.289	0.291	0.287	0.288
3	2570.2	2573.3	2531.7	2565.2	2553.9	2554.9	2557.2	0.285	0.283	0.280	0.283	0.284	0.281	0.282
4	2567.8	2568.8	2505.0	2561.6	2537.9	2542.8	2547.0	0.279	0.276	0.270	0.276	0.275	0.273	0.275
5	2531.2	2554.8	2492.2	2541.3	2514.0	2531.6	2529.5	0.269	0.271	0.264	0.269	0.267	0.268	0.268
6	2527.8	2546.2	2481.9	2534.1	2509.1	2523.4	2523.2	0.267	0.268	0.260	0.267	0.264	0.265	0.265
7	2523.1	2549.5	2479.5	2533.1	2507.2	2524.6	2522.7	0.260	0.263	0.254	0.261	0.258	0.260	0.260
8	2535.5	2544.3	2474.9	2536.5	2513.2	2517.2	2523.5	0.259	0.260	0.252	0.259	0.257	0.257	0.258
9	2510.1	2516.1	2421.3	2506.6	2468.5	2480.3	2485.8	0.253	0.252	0.238	0.251	0.246	0.247	0.248
10	2514.2	2518.5	2426.9	2508.0	2475.4	2487.5	2491.4	0.250	0.250	0.237	0.249	0.244	0.246	0.247
11	2518.9	2530.1	2411.9	2513.6	2472.1	2490.3	2492.4	0.248	0.249	0.231	0.247	0.241	0.243	0.244
12	2492.2	2534.9	2410.5	2512.3	2463.7	2493.5	2491.7	0.240	0.245	0.228	0.243	0.236	0.239	0.240
13	2507.1	2550.2	2418.0	2528.0	2474.8	2502.9	2504.4	0.239	0.244	0.225	0.241	0.235	0.237	0.238
14	2520.8	2557.9	2425.3	2539.8	2488.6	2511.6	2516.3	0.239	0.243	0.223	0.241	0.234	0.235	0.237

Table S.7: AIC and MSE under different combinations of p and $\mathbf{W}$ for $d_0 = 6$.

	AIC							MSE						
p	$\mathbf{W}_A$	$\mathbf{W}_B$	$\mathbf{W}_U$	$\mathbf{W}_{AB}$	$\mathbf{W}_{AU}$	$\mathbf{W}_{BU}$	$\mathbf{W}_{ABU}$	$\mathbf{W}_A$	$\mathbf{W}_B$	$\mathbf{W}_U$	$\mathbf{W}_{AB}$	$\mathbf{W}_{AU}$	$\mathbf{W}_{BU}$	$\mathbf{W}_{ABU}$
1	2791.9	2802.4	2699.3	2777.0	2738.7	2752.7	2750.4	0.323	0.320	0.308	0.317	0.315	0.313	0.314
2	2629.7	2626.1	2577.5	2615.9	2603.6	2598.3	2602.5	0.294	0.290	0.286	0.290	0.291	0.287	0.289
3	2575.4	2577.5	2530.5	2569.0	2554.7	2556.4	2559.1	0.287	0.284	0.280	0.284	0.284	0.282	0.283
4	2574.7	2575.5	2505.8	2567.9	2540.6	2547.0	2551.4	0.280	0.278	0.270	0.277	0.276	0.274	0.275
5	2539.2	2563.9	2494.9	2549.6	2518.3	2538.2	2536.0	0.270	0.272	0.265	0.271	0.268	0.269	0.269
6	2535.4	2554.6	2484.5	2541.7	2513.1	2529.3	2529.0	0.268	0.270	0.261	0.268	0.265	0.266	0.266
7	2530.7	2558.6	2481.5	2541.1	2511.1	2530.3	2528.4	0.262	0.265	0.255	0.263	0.259	0.260	0.261
8	2542.2	2553.7	2476.9	2543.8	2516.4	2523.0	2528.8	0.261	0.261	0.252	0.260	0.257	0.257	0.258
9	2516.1	2525.2	2422.3	2513.4	2471.0	2485.7	2490.7	0.253	0.253	0.238	0.252	0.246	0.247	0.249
10	2520.9	2527.8	2429.1	2515.3	2479.2	2493.1	2496.9	0.250	0.251	0.236	0.249	0.244	0.246	0.247
11	2524.4	2538.4	2413.5	2520.0	2475.0	2495.2	2497.2	0.248	0.250	0.231	0.248	0.241	0.243	0.244
12	2496.6	2542.3	2410.6	2517.7	2465.2	2497.5	2495.3	0.240	0.246	0.228	0.243	0.236	0.239	0.240
13	2511.0	2556.5	2418.3	2532.5	2476.6	2506.6	2507.8	0.239	0.244	0.225	0.242	0.235	0.237	0.238
14	2523.8	2562.9	2424.9	2543.2	2489.5	2514.2	2518.6	0.239	0.243	0.222	0.241	0.233	0.235	0.237

Table S.8: AIC and MSE under different combinations of p and $\mathbf{W}$ for $d_0 = 7$.

	AIC							MSE						
p	$\mathbf{W}_A$	$\mathbf{W}_B$	$\mathbf{W}_U$	$\mathbf{W}_{AB}$	$\mathbf{W}_{AU}$	$\mathbf{W}_{BU}$	$\mathbf{W}_{ABU}$	$\mathbf{W}_A$	$\mathbf{W}_B$	$\mathbf{W}_U$	$\mathbf{W}_{AB}$	$\mathbf{W}_{AU}$	$\mathbf{W}_{BU}$	$\mathbf{W}_{ABU}$
1	2794.9	2807.2	2707.7	2781.5	2744.6	2759.6	2756.5	0.321	0.319	0.308	0.316	0.314	0.313	0.313
2	2632.1	2628.5	2581.3	2618.1	2606.8	2601.3	2605.3	0.293	0.289	0.286	0.289	0.290	0.287	0.288
3	2578.0	2579.8	2532.7	2571.1	2557.0	2558.6	2561.3	0.286	0.283	0.280	0.283	0.284	0.281	0.282
4	2577.0	2577.2	2507.5	2569.7	2542.5	2548.8	2553.3	0.280	0.277	0.270	0.277	0.275	0.273	0.275
5	2540.7	2565.0	2495.3	2550.7	2518.7	2538.9	2536.7	0.270	0.272	0.264	0.270	0.268	0.269	0.269
6	2536.6	2555.4	2484.7	2542.3	2513.2	2529.6	2529.3	0.268	0.269	0.261	0.268	0.265	0.265	0.266
7	2531.8	2559.4	2481.5	2541.6	2510.9	2530.2	2528.4	0.262	0.265	0.254	0.262	0.258	0.260	0.260
8	2542.7	2554.5	2477.3	2544.0	2516.1	2523.0	2528.6	0.260	0.261	0.252	0.260	0.257	0.257	0.258
9	2516.0	2525.7	2422.2	2513.1	2470.0	2484.7	2489.7	0.253	0.252	0.237	0.251	0.245	0.246	0.248
10	2521.1	2527.1	2429.1	2514.1	2477.9	2491.0	2495.0	0.250	0.250	0.236	0.249	0.243	0.245	0.246
11	2524.2	2537.5	2414.0	2518.3	2473.6	2493.1	2494.9	0.248	0.249	0.230	0.247	0.240	0.242	0.243
12	2497.7	2541.1	2412.6	2516.0	2465.2	2495.2	2493.3	0.240	0.245	0.227	0.242	0.236	0.238	0.239
13	2511.7	2555.0	2419.8	2530.4	2476.1	2503.9	2505.3	0.239	0.243	0.225	0.241	0.234	0.236	0.237
14	2525.4	2561.8	2426.6	2541.3	2490.0	2511.9	2516.6	0.238	0.242	0.222	0.240	0.233	0.234	0.236

Table S.9: AIC and MSE under different combinations of p and $\mathbf{W}$ for $d_0 = 8$.
